# L-selectin: A Major Regulator of Leukocyte Adhesion, Migration and Signaling

**DOI:** 10.3389/fimmu.2019.01068

**Published:** 2019-05-14

**Authors:** Aleksandar Ivetic, Hannah Louise Hoskins Green, Samuel James Hart

**Affiliations:** King's College London, School of Cardiovascular Medicine and Sciences, BHF Center of Research Excellence, London, United Kingdom

**Keywords:** L-selectin (CD62, SELL), migration, inflammation, signaling, adhesion, monocyte, lymphocyte, Neutrophil (PMN)

## Abstract

L-selectin (CD62L) is a type-I transmembrane glycoprotein and cell adhesion molecule that is expressed on most circulating leukocytes. Since its identification in 1983, L-selectin has been extensively characterized as a tethering/rolling receptor. There is now mounting evidence in the literature to suggest that L-selectin plays a role in regulating monocyte protrusion during transendothelial migration (TEM). The N-terminal calcium-dependent (C-type) lectin domain of L-selectin interacts with numerous glycans, including sialyl Lewis X (sLe^x^) for tethering/rolling and proteoglycans for TEM. Although the signals downstream of L-selectin-dependent adhesion are poorly understood, they will invariably involve the short 17 amino acid cytoplasmic tail. In this review we will detail the expression of L-selectin in different immune cell subsets, and its influence on cell behavior. We will list some of the diverse glycans known to support L-selectin-dependent adhesion, within luminal and abluminal regions of the vessel wall. We will describe how each domain within L-selectin contributes to adhesion, migration and signal transduction. A significant focus on the L-selectin cytoplasmic tail and its proposed contribution to signaling via the ezrin-radixin-moesin (ERM) family of proteins will be outlined. Finally, we will discuss how ectodomain shedding of L-selectin during monocyte TEM is essential for the establishment of front-back cell polarity, bestowing emigrated cells the capacity to chemotax toward sites of damage.

## L-Selectin Gene Expression

**Figure 1:**
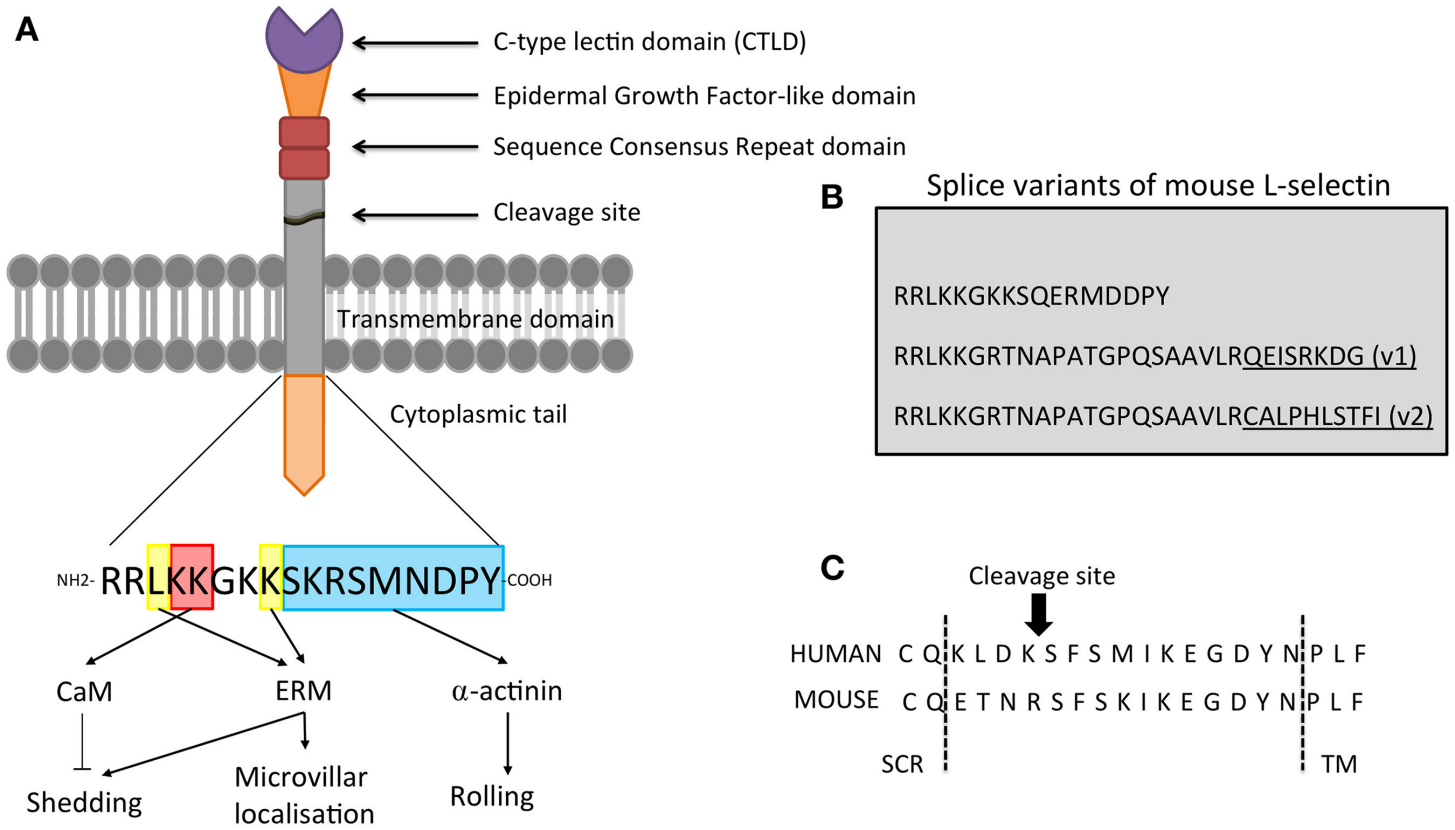
Domain organization of L-selectin. **(A)** L-selectin is a type I transmembrane glycoprotein. Going from N-terminal to C-terminal, it is broken down into: C-type lectin domain (CTLD), Epidermal Growth Factor (EGF)-like domain, two sequence consensus repeat (SCR) domains, a cleavage site, transmembrane domain and a 17 amino acid cytoplasmic tail. Amino acid sequence of the cytoplasmic tail of human L-selectin is depicted, highlighting the amino acids that support binding to calmodulin (CaM), ERM proteins, and alpha-actinin. **(B)** The three sequences correspond to the mouse L-selectin tail (note the mouse L-selectin tail has a single serine at position 364, whereas the tail of human L-selectin possesses an extra serine residue at position 367). Sequence conservation at the membrane-proximal region that support binding to ERM and CaM (RRLKKG) is 100% conserved. The amino acid sequences of two splice variants of mouse L-selectin (v1 and v2) are provided in the sequences below. Underlined residues represent sequences that are unique to v1 and v2. **(C)** Amino acid sequences surrounding the cleavage site of human and mouse L-selectin. Arrow indicates the position of cleavage. TM, transmembrane domain; SCR, sequence consensus repeat region.

The human L-selectin gene (*sell*) is located on the long arm of chromosome 1 (1q24.2), and is arranged in tandem with its family members (in the order: L-, P-, and E-selectin). L-selectin consists of ten exons spanning a region of ~21.0 kb. FOXO1 regulates transcription of the human *sell* gene (1, 2), whereas chromosome immunoprecipitation experiments identify other transcription factors (Mzf1, Klf2, Sp1, Ets1, and Irf1) in regulating the mouse *sell* gene (3). Splicing of the exons into mature mRNA is translated to form a protein product with a predicted molecular weight of 30 kDa. However, the actual molecular weight of L-selectin differs between cell types—ranging from 65 kDa in lymphocytes to 100 kDa in neutrophils and is due to cell type-specific glycosylation (4–10). It is highly likely that altered glycosylation patterns in L-selectin could dictate cell-specific functions, but this has not been explored in any detail. L-selectin is organized into distinct structural domains: a ligand binding calcium-dependent (C-type) lectin domain (CTLD), an EGF-like domain, two complement-like repeat sequences and an extracellular cleavage site (Figure 1A).

### Splice Variants of L-selectin

Splice variants of L-selectin have been identified and characterized in both mice (11) and humans (12). The mouse *sell* gene is composed of 9 exons. The two splice variants, termed L-selectin-v1 and L-selectin-v2, possess an additional exon, nested between exons 7 and 8. The splice variants share the first 49bp sequence, whereas L-selectin-v2 extends for an extra 51bp that is immediately 3' to this region. Both splice variants possess longer cytoplasmic tails (WT = 17 aa; v1 = 30 aa; v2 = 32aa—see Figure 1B). The overall mRNA levels of L-selectin-v1 and -v2 constitute 2–3% of the total L-selectin mRNA, so its impact in endogenous leukocyte trafficking and signaling is not fully understood. However, over-expression of these variants in cells lacking L-selectin reveal altered capacities in adhesion to sLe^x^ under flow, ectodomain shedding in response to cellular activation, and signaling to p38 MAPK following antibody-mediated clustering (AMC) (11).

The human splice variant lacks exon 7, which codes for the transmembrane domain (12), so transcripts lacking this exon are secreted and soluble. Patients with rheumatic disease presented an increased prevalence of the splice variant transcript, which is thought to contribute to the increase in soluble L-selectin. Based on the relatively low abundance of the variant transcript, it is currently not clear what proportion of circulating soluble L-selectin is represented by either the cleaved form (e.g., through “basal shedding”—see later) or the spliced transmembrane-less forms.

## Regulation of L-Selectin Protein Expression in Divergent Leukocyte Subsets

The identification of human neonatal bone marrow CD10^−^CD62L^hi^ cells reveals that L-selectin is one of the earliest surface markers to be expressed on lymphoid-primed hematopoietic stem cells (13). Similar observations have been made in mice, suggesting L-selectin plays a pivotal role in stem cell trafficking and differentiation (14, 15). CD10^−^CD62L^hi^ cells can differentiate into B cells, dendritic cells, monocytes, NK cells and T cells. It is likely that the L-selectin expressed in these early progenitor cells is required for trafficking from the bone marrow toward peripheral lymphoid organs. Whether L-selectin-dependent signaling also contributes to subsequent differentiation is not known.

The average leukocyte will express ~50,000–70,000 molecules of L-selectin at the plasma membrane (16, 17). Numerous reports have shown that L-selectin is anchored on finger-like projections called microvilli, which increases tethering efficiency during recruitment (18–20). Protein expression of L-selectin is constitutive on most circulating leukocytes, and is slowly turned over at the plasma membrane through a process of ectodomain shedding (commonly referred to as “shedding”). A variety of artificial or physiological agonists of cell activation can, within minutes, promote robust L-selectin shedding in numerous leukocyte sub-types (21–24). The zinc-dependent metalloproteinase, a disintegrin and metalloproteinase (ADAM)17, is the major enzyme responsible for L-selectin shedding in leukocytes (25–28). However, ADAMs 8 and 10 have also been reported to cleave L-selectin in specific settings (29, 30). From a clinical perspective, the soluble circulating form of L-selectin (released as a consequence of ectodomain shedding) is sometimes used as a surrogate plasma/serum biomarker for leukocyte activity triggered during acute or chronic inflammation (31–35). Similarly, leukocytes expressing low levels of surface L-selectin (judged by flow cytometric analysis) are a classic indicator of cellular activation (24, 34). Paradoxically, a drop in soluble L-selectin can also be detected in certain diseases, such as sepsis (36). The drop in detectable soluble L-selectin could be due to its adsorption to luminal vascular ligands that are upregulated during sepsis. Alternatively, L-selectin could be cleaved from transmigrated neutrophils within abluminal/non-luminal regions of vessels (see later). Although soluble L-selectin can compete for cell-associated L-selectin, little is understood about how the two forms buffer leukocyte recruitment during inflammation. Soluble L-selectin is detected in the plasma of healthy humans (0.7-−1.5 μg per mL), suggesting cell-associated L-selectin is cleaved from resting circulating leukocytes at low levels. Indeed, mouse neutrophils lacking ADAM17 express higher than average surface levels of L-selectin (26, 37). This phenomenon is also observed when broad-spectrum synthetic inhibitors of ADAM17 are used, or when the cleavage site of L-selectin is mutated and rendered “sheddase-resistant” (38–40). The manner in which L-selectin expression is regulated, either at the translational or post-translational level, will be unique in different leukocyte subsets. For example, the lifespan of a central memory T-cell (T^CM^) far exceeds the lifespan of a neutrophil (i.e., 6 weeks compared to 8 days, respectively) (41, 42). Aging neutrophils possess waning levels of L-selectin, which contrasts with T^CM^ (43–45). The sections below provide a few examples of how L-selectin is expressed in different leukocyte subsets and the impact that this might have on immune cell behavior.

### The Impact of L-selectin Expression in Lymphocytes

Lymphocytes constitute ~78–88% of the total white blood cell count in mice. These values are quite different in humans (20–40%). L-selectin-deficient mice expose the absolute requirement for L-selectin in the trafficking of naïve T-cells to lymph nodes (46). Immunofluorescence imaging of frozen mouse lymph node sections reveals that L-selectin is cyclically expressed in recirculating naïve T-cells as they adhere to and extravasate across high endothelial cell (HEC) monolayers and enter the lymph node parenchyma (47). These observations would suggest ectodomain shedding is triggered during adhesion and TEM, and that re-expression of L-selectin occurs upon their entry into the lymphatics, via the thoracic duct. Increasing L-selectin expression in naïve T-cells does not enhance homing of T-cells to lymph nodes, suggesting that L-selectin densities can reach saturation within specific plasma membrane domains (e.g., on microvillar tips) (48).

Signaling downstream of the T-cell receptor triggers the activation of PI3Kδ, which leads to ectodomain shedding and concomitant inhibition of Klf2-dependent expression of L-selectin in mouse T-cells (49). The terminal differentiation of naïve T-cells into effector memory T-cells (T^EM^) involves both L-selectin shedding and the transcriptional shutdown of the L-selectin gene. Collectively, these events drive the trafficking of T^EM^ away from secondary lymphoid organs toward peripheral tissues (39, 40). T^CM^ are smaller in size and have longer telomere lengths than T^EM^, but it is not clear if the respective presence or absence of L-selectin expression contributes to the phenotype (45). Expression of L-selectin in T^CM^ is essential for trafficking toward, and the immune surveillance of, peripheral lymph nodes (39, 40).

Regulatory T-cells (Tregs) play important immunosuppressive roles in diverse immune responses in health and disease [e.g., graft-vs.-host disease (50)]. Tregs are characterized by the presence of the transcription factor FoxP3, which can be further subdivided according to the expression of surface epitopes, including L-selectin. Tregs expressing L-selectin have been shown to have superior immunosuppressive roles in different disease models (51–54). Again, it is not clear if L-selectin-dependent trafficking and/or signaling contributes to improved immunosuppressive function.

The surface level of L-selectin is significantly reduced in naïve CD4 T-cells following HIV entry (55–59). The HIV accessory protein, Vpr, can increase L-selectin mRNA expression in primary human naïve T-cells and Jurkat T-cells, but its impact on the pathobiology of HIV is unclear (59). In contrast, the other accessory proteins, Nef and Vpu, can sequester L-selectin into subcellular compartments and decrease cell surface expression (57, 60). Such post-translational mechanisms of reducing L-selectin surface expression by HIV is thought to prevent trafficking back to secondary lymphoid organs, redirecting infected T-cells to the systemic circulation to increase viremia. L-selectin expression on naive CD4 T-cells has recently been defined as a novel receptor for HIV entry (58), and work from this study reveals that L-selectin shedding ensues 4–6 days post-infection. Moreover, HIV preferentially infects T^CM^ over T^EM^ cells – positively correlating with L-selectin expression. Blocking ectodomain shedding of L-selectin with (broad-spectrum) synthetic inhibitors to ADAM17 (BB-94, TAPI-1, TAPI-2) does not increase HIV attachment or entry, but reduces the release of viral particles (58). These findings suggest that blocking ectodomain shedding of L-selectin may alter membrane/cytoskeleton dynamics that are dependent on the release of HIV particles from infected T-cells.

One study has recently shown that increasing L-selectin expression in cytotoxic CD8 T-cells is causal to viral clearance (61). L-selectin expression is rapidly lost when antigen-presenting cells prime naïve recirculating CD8 T-cells in lymph nodes. Re-expression of L-selectin is detected when antigen-primed CD8 T-cells egress the lymph node, which is now known to be essential for trafficking toward visceral or mucosal virus-infected organs. Bestowing antigen-primed CD8 T-cells with higher levels of L-selectin, by genetically rendering the molecule non-cleavable, increases clearance of viral-infected sites without obvious signs of altering cytokine secretion profiles or clonal expansion. Interestingly, L-selectin shedding in tumor antigen-primed human CD8 T^CM^ cells inversely correlates with the upregulation of the degranulation marker, CD107a, and enhanced tumor lytic activity (62). It is not exactly clear how L-selectin contributes to tumor or virus killing, and whether conserved mechanisms exist between mice and humans. Previous studies have shown that L-selectin clustering can augment T-cell receptor signaling, suggesting roles beyond just trafficking (63, 64).

Although L-selectin can act as a co-stimulator of specific leukocytic responses, it is much harder to model this experimentally without the prior stimulation of L-selectin—e.g., via AMC. In this regard, AMC of L-selectin on mouse splenic T-cells (CD4 and CD8) and B-cells can increase their responsiveness to the chemokine CCL21, via CCR7 (65). Interestingly, the density of surface-expressed L-selectin within different T-cell subsets (i.e. CD62L^lo^CD44^hi^ [T^EM^] or CD62L^hi^CD44^lo^ [T^CM^] subsets within CD4 cells, or CD8 cells) positively correlated with enhanced chemotaxis toward CCL21. This model suggests that clustering of L-selectin by endothelial expressed ligands (e.g., within HEV) can facilitate the transmigration of T-cells into lymph nodes. Spleen tyrosine kinase (Syk) was shown in this study to have *in vivo* importance in this mechanism.

Manipulating cell surface levels of L-selectin on leukocytes can impact the pathogenesis of atherosclerosis. Mice lacking L-selectin will develop accelerated atherosclerosis (66), suggesting that L-selectin-dependent trafficking to atherosclerotic lesions is somehow protective. Flow cytometric phenotyping of immune cell infiltrates within aortae of CD62L^+/+^ApoE^−/−^ and CD62L^−/−^ApoE^−/−^ mice, revealed that two B-cell subsets, B1a and Bregs, provided an anti-inflammatory role in the pathogenesis of atherosclerosis (67). As atherosclerotic lesions mature, the microvascular network of the vasa vasorum can innervate into late developing lesions – acting as a portal for increased leukocyte trafficking (via “the back door”) into lesions (68). Moreover, the increased microvascular density within late developing regions is deemed a predicator for unstable plaque rupture (69, 70). Endothelial cells lining the microvessels within these late lesions were shown to upregulate P-selectin proteoglycan ligand-1 (PSGL-1), a known ligand for L-selectin (68). The data, collectively, suggests that L-selectin-dependent recruitment of leukocytes into early atherosclerotic lesions is protective, but, as the disease progresses, L-selectin-dependent recruitment of leukocytes can be pathogenic.

### Regulation of L-selectin Expression in Neutrophils

Human neutrophils represent 50–70% of the total white blood cell count, which contrasts with values of 10–25% in mice (71). L-selectin regulates neutrophil trafficking to sites of inflammation, but our understanding of the intracellular mechanisms underpinning this process is still incomplete. The first *in vitro* transmigration experiments involved the addition of primary human neutrophil suspensions onto cytokine-activated monolayers of human umbilical vein endothelial cells (HUVEC). In such assays, L-selectin is rapidly cleared from the neutrophil plasma membrane through ectodomain shedding (72). L-selectin shedding is a classic readout for neutrophil activation and/or “priming” (i.e., partial activation), which is concordant with an upregulation in CD11b expression (αMβ2 or Mac-1) (24). Blocking L-selectin shedding in human neutrophils with broad-spectrum inhibitors for ADAM17 (e.g. Ro-31- 9790, KD-IX-73-3, TAPI-0, TAPI-1, TMI005, and GM6001) does not impact rolling velocity or the kinetics of TEM (72), which is contrary to what has been observed in mice (73).

AMC of L-selectin on human neutrophils can result in its own ectodomain shedding (74), acting as a self-limiting mechanism by which L-selectin can behave as a cell adhesion molecule and signaling receptor. Prolonged perfusion of neutrophils over immobilized sLe^x^ can increase rolling velocity over time, which is directly due to L-selectin shedding. The activation of p38 MAPK drives L-selectin shedding during rolling, and the term “mechanical shedding” was coined to explain this phenomenon (75). Mechanical shedding has only been characterized in human neutrophils rolling on sLe^x^, but blocking L-selectin shedding in mice with KD-IX-73-3 also reduces neutrophil rolling velocities (73, 76). These studies collectively indicate that mechanical shedding could have *in vivo* significance. However, it should be noted that L-selectin on human neutrophils is decorated itself with sLe^x^, acting as a ligand for E-selectin (8, 9), so this would invariably lead to altered mechanisms of L-selectin-dependent recruitment of human neutrophils (see later).

A recent study has challenged the view that L-selectin shedding is a classic outcome of neutrophil transmigration (77). Neutrophils harvested from synovial fluid of arthritic patients registered L-selectin positive and showed little signs of priming. L-selectin shedding is therefore not a strict prerequisite for neutrophil emigration into inflamed tissue. Interestingly, neutrophils aspirated from the fluid of acutely formed skin blisters were shown to be primed (e.g., CD11b-positive) and L-selectin-negative. These observations suggest that the mechanisms of neutrophil migration toward acute or chronic inflammation could differ in the expression and turnover of adhesion molecules. Indeed, chemokine receptor expression on the neutrophils and the array of cytokines and chemokines in synovial vs. blister fluid microenvironments appear to be radically different (77).

L-selectin-null mice display significant reductions in neutrophil recruitment to a 4 h model of thioglycollate-induced peritonitis (46). Monitoring the rolling flux of neutrophils in post capillary venules of exteriorised cremasteric muscle (induced by surgical trauma) showed no differences between L-selectin-null mice and WT controls at early time points. However, a significant reduction in neutrophil rolling flux of L-selectin-deficient neutrophils was detected after 50 min of cremaster exteriorisation. Superfusing exteriorised cremasteric tissue with platelet activating factor (PAF), a potent neutrophil chemoattractant, showed only modest reductions in the rolling flux of L-selectin-null neutrophils (78). This observation supports the idea that L-selectin ligands might be absent in endothelial cells lining cremasteric post-capillary venules (79). Although no difference in neutrophil adhesion to post-capillary venules of either WT or L-selectin-null mice was observed, neutrophil emigration from post-capillary venules was significantly reduced in neutrophils lacking L-selectin. Moreover, interstitial chemotaxis toward a gradient of the chemokine CXCL1 (or Keratinocyte-derived Chemoattractant (KC)—another potent neutrophil chemoattractant) was severely impaired in the small proportion of emigrated neutrophils (78). These findings led to the hypothesis that L-selectin may regulate neutrophil chemotaxis, suggesting extended roles beyond tethering and rolling. Neither of these studies explored whether L-selectin was shed in response to PAF superfusion or chemotaxis toward KC. Interestingly, subjecting neutrophils that express non-cleavable L-selectin to a model of KC-induced interstitial chemotaxis phenocopied the behavior witnessed in neutrophils lacking L-selectin. These results seem counterintuitive, but suggest that the membrane-retained fragment (MRF - i.e., the “stump” of L-selectin retained after shedding) may be responsible for guiding neutrophil interstitial chemotaxis, as the MRF can only be processed from WT L-selectin.

The migration of circulating neutrophils into the inflamed peritoneum results in a significant reduction in L-selectin expression, matching what has been observed *in vitro* with human neutrophils crossing activated HUVEC monolayers (72). Knock-out studies reveal that this mechanism is through ectodomain shedding via ADAM17 (26). Mouse neutrophils lacking ADAM17 express 10 times more surface L-selectin, but will still lose a significant amount of this when entering the peritoneum (26). Whether the observed loss is through the action of a related ADAMs protease member (e.g., ADAM10 or ADAM8) (29, 30) or through the loss of membrane fragments (e.g., exosomes) has not been fully determined. The higher L-selectin expression in ADAM17-null neutrophils drives slower rolling velocities in inflamed cremasteric post-capillary venules.

Neutrophil aging in the circulation leads to the progressive increase in CXCR4 expression, with a corresponding decrease in L-selectin expression (80). An increase in CXCR4 expression is required for homing of aged neutrophils to the bone marrow for macrophage-mediated clearance (81). However, it is not clear if the reduced L-selectin expression is causal to, or an epiphenomenon of, neutrophil aging. Furthermore, it is not clear if the reduction in L-selectin expression is at the translational or post-translational level (e.g., increased basal shedding or exosome-like mediated release).

The impact of L-selectin expression on neutrophil behavior has been extensively reviewed elsewhere (82).

### L-selectin in Monocytes

Monocytes constitute ~2–8% of all circulating white blood cells in the adult human, which contrasts with 0.9–1.5% in mice. Within the human population, a further three monocyte subsets are characterized according to the relative abundance of two surface markers: CD14^++^ CD16^−^ or “classical” (80–95%), CD14^++^ CD16^+^ or “intermediate” (2–11%), and CD14^+^ CD16^++^ or “non-classical” (2–8%). Classical monocytes express high levels of L-selectin, which is in stark contrast to the lesser abundant intermediate and non-classical monocytes (83).

L-selectin mediates the recruitment of human monocytes to activated endothelial monolayers under flow conditions (84, 85). Subsequent experiments showed that L-selectin/PSGL-1 interactions in trans are responsible for the adhesion of monocytes through the process of secondary tethering and rolling (i.e., the interaction of bystander leukocytes with already adherent leukocytes) (86). Imaging of L-selectin on classical monocytes crossing TNF-α-activated endothelial monolayers shows that L-selectin shedding is triggered specifically during TEM and not during earlier phases of the multi-step adhesion cascade (87). These observations challenge current perceptions of L-selectin shedding occurring during the phase of “firm adhesion” in the multi-step adhesion cascade. Two recent articles suggest L-selectin plays a role in regulating monocyte pseudopod protrusion during TEM (87, 88). Interestingly, expression of L-selectin in monocyte-like THP-1 cells (which do not express endogenous L-selectin) bestows them with a more invasive phenotype (i.e., possessing a higher propensity to protrude through inflamed endothelial monolayers) (87, 88).

The secreted mucin AgC10, released from the parasite *Trypanosoma cruzi*, can bind to L-selectin on human monocytes, which over a 4–6 h period can induce a reduction in L-selectin expression via ectodomain shedding (89). Consequently, monocytes treated with AgC10 have a reduced capacity to interact with activated endothelial monolayers under shear stress conditions.

The equivalent of classical monocytes in mice is characterized by: CX3CR1^low^, CCR2^+^, Ly6C^+^, and CD26L^+^. On the other hand, “patrolling monocytes” are considered to be the mouse equivalent of non-classical monocytes (CX3CR1^hi^, CCR2^−^, Ly6C^−^, CD62L^−^), which will typically remain in the circulation and patrol the endothelium for infection and/or damage (90). In both human and mice, the classic monocytes share L-selectin and CCR2 expression. It would be interesting to test if the expression of L-selectin in patrolling monocytes would bestow a more pro-invasive phenotype.

Invading human cytotrophoblasts, which are tasked to bridge a gap between the placenta and the uterus, are thought to employ L-selectin to mediate interstitial migration (91, 92). These findings further support extended roles for L-selectin beyond classic leukocyte tethering and rolling—moreover within microenvironments that are devoid of hydrodynamic shear stress.

Using a mouse model of thioglycollate-induced peritonitis, researchers showed that the turnover of L-selectin is radically different between monocytes and neutrophils: L-selectin expression drops in emigrated neutrophils, but remains unchanged in monocytes (26). The differences underpinning these L-selectin dependent and independent modes of leukocyte emigration are not understood, nor are their relevance to human monocyte TEM (87, 88).

The exposure of isolated human monocytes to selenium promotes metalloproteinase-dependent shedding of L-selectin (93), leading to reduced adhesion on immobilized ligand under flow conditions. Furthermore, the observed shedding could be reversed using synthetic inhibitors of ADAM17 (i.e., GM6001). Administering selenium to mice led to a significant increase in soluble circulating L-selectin. Reducing agents such as dithiothreitol have been shown to increase ADAM17 activity (94), but whether the anti-oxidant properties of selenium could be directly/indirectly linked to reducing cysteines on ADAM17 has not been explored.

## Domain Organization of L-Selectin

L-selectin shares a number of common extracellular domains with its family members, E-, and P-selectin (Figure 1A). In contrast, the cytoplasmic tails of the selectin family members bear no resemblance to one another and likely transduce unique intracellular signals. In this section we will discuss the roles that each domain plays with respect to adhesion, signaling, and ectodomain shedding.

### CTLD and the EGF-Like Domain

L-selectin possesses an N-terminal CTLD (95), which belongs to a large superfamily of metazoan proteins with diverse functions (96, 97). The CTLD of L-selectin directly regulates leukocyte tethering and rolling by interacting with the minimal determinant tetrasaccharide, sLe^x^ (98). A unique feature of the selectins is to stabilize bond lifetimes through conformational changes in the CTLD that occurs in response to an external force, such as hydrodynamic shear stress. Mutagenesis of E-selectin showed the existence of coordinate bonds between Ca^2+^ and amino acid residues within the upper face of the CTLD stabilizes the interaction (99, 100). Note that these residues are also common to L-selectin. The 3- and 4-hydroxyl groups of fucose in sLex also form a coordinate bond to Ca^2+^, which collectively stabilize selectin/ligand binding (101). Binding of L-selectin to sLe^x^ occurs within a critical threshold of shear stress (typically between 0.3 and 1.0 dynes per cm^2^). L-selectin binding obeys a ‘catch' and ‘slip' bond mechanism, where optimal shear stress conditions can expose more of the ligand-binding domain and increase adhesiveness. The bond ‘slips' as the tethered cell maneuvers over the ligand-bound site, where an increase in tensile force eventually exceeds the threshold of the catch-bond (102). The catch-slip bond phenomenon is described in more detail elsewhere, elaborating on the existence of a triphasic (slip-catch-slip) behavior of binding (103–105).

X-ray crystallographic evidence of the selectins reveals that the CTLD folds onto a region of the EGF-like domain, linked by a hydrogen bond between Y37 and N138 (101, 106). This inter-domain interaction constrains L-selectin into a less adhesive conformation whilst in the circulation. The functional significance of this interaction was characterized in neutrophils, where uncoupling the Y37/N138 hydrogen bond increases the bond lifetime, which manifests in increased neutrophil priming within the circulation (107). Neutrophils bearing an N138G knock-in mutation within L-selectin displayed enhanced bacterial killing and worsened outcomes in models of sterile injury. Both of these phenotypes were directly linked to the increased priming of neutrophils, confirming causality to the knock-in mutation. It has been suggested that this mode of “mechanochemistry” exists in other immune cells that express L-selectin (107).

### SCR Domains

All three selectin family members possess a varying number of SCRs (in humans L-selectin has 2, E-selectin has 6, and P-selectin has 9), which bear homology to complement regulatory proteins. SCRs are also termed “sushi domains” and are present in a number of different cell adhesion molecules (108). In respect of the selectins, the SCR serves to distance the CTLD from the plasma membrane, placing it in a strategically advantageous position, reaching out beyond other cell adhesion molecules, to support tethering and rolling behavior. L-selectin is thought to have fewer than its family members as it is anchored to microvilli, which bestows advantageous positioning for tethering under flow.

### Cleavage Domain

L-selectin undergoes ectodomain shedding at a specific membrane-proximal location, eleven amino acids above the transmembrane domain—between positions K321 and S322 (109) (see Figure 1C). Alanine scanning mutations surrounding the cleavage site would suggest that there is redundancy in ADAM17 recognition of the cleavage site. Deletion of multiple amino acids suggests that the actual distance of the cleavage site from the plasma membrane is more important (110). Studies in ADAM17 knock-out mice reveal the accumulation of L-selectin on the surface of neutrophils and monocytes, strongly suggesting that both the turnover and induced ectodomain shedding is mediated by a similar enzyme. Due to the relaxed specificity of the L-selectin cleavage site, it is difficult to definitively state that both basal and activated shedding target K321/S322. Binding of calmodulin to the L-selectin cytoplasmic domain is thought to regulate the conformation of the cleavage site (see later), where binding confers protection from shedding and dissociation renders susceptibility to proteolytic attack (111).

### Transmembrane Domain

Swapping the transmembrane domain of L-selectin with that of CD44 has been shown to alter the subcellular location away from microvilli and toward the cell body, suggesting that amino acids within this region are responsible for the anchoring L-selectin to microvilli (112, 113). It's currently not understood what the amino acids within the transmembrane domain might interact with to retain L-selectin in microvilli, but intramembrane proteases [such as presenilins (114)] could be involved. For example, CD44 is a known target of γ-secretase (115–117), the active site of which is resident within the plasma membrane. It's currently not known if γ-secretases can bind to CD44 without cleaving it and whether its interaction could influence its subcellular localization on the plasma membrane. More subtle mutations within the L-selectin tail, which have been shown to abrogate ERM binding, can phenocopy the microvillar displacement (20).

### Cytoplasmic Tail

The tail of L-selectin is only 17 amino acids long, yet it has been documented to bind up to 6 intracellular proteins, which include: alpha-actinin (118), calmodulin (111), ezrin, moesin (119), protein kinase C (PKC) isozymes (120) and μ1alpha/AP-1 (121). Given the size of the L-selectin tail, not all of these proteins can bind simultaneously, but are likely to interact under tight spatio-temporal constraints—e.g., during tethering, rolling, firm adhesion, and TEM. More of this will be discussed in the section below.

Deleting the C-terminal 16 amino acids of the L-selectin tail can dramatically impact its lateral mobility along the plane of the plasma membrane, brought about by a lack of anchoring to the underlying cortical actin-based cytoskeleton (122). Such large truncations can disrupt tethering dynamics under flow conditions (123) and reduces the efficiency of L-selectin shedding (38, 110, 124).

The L-selectin tail is highly basic, with a pI of approximately 11.0, increasing its attraction toward negatively charged phospholipids within the inner leaflet of the plasma membrane. Recent evidence, both *in vitro* and *in silico*, support the view that the tail of L-selectin folds onto the inner leaflet of the plasma membrane to form an “L-shaped configuration” (125–127). Binding of ERM proteins to the tail of L-selectin is thought to desorb the tail from the inner leaflet of the plasma membrane and render it competent for calmodulin binding (126).

## Binding Partners of the L-Selectin Tail

Numerous proteins have been shown to co-precipitate with L-selectin in biochemical experiments (mainly through standard immunoprecipitation or “pull-down” techniques). These observations do not imply direct binding, but may represent proteins belonging to higher-ordered complexes, and would require further validation by interdisciplinary means. As mentioned above, the cytosolic tail of L-selectin is highly basic and can attract false-positive binders from whole cell lysates. Experiments using the L-selectin tail peptide should undertake a “pre-clearing” step, where the exposure of the cell lysate to a scrambled form will attract false binders based on charge without sequence specificity. The sections immediately below list known and validated binding partners of the L-selectin tail.

### Alpha-Actinin

Alpha-actinin is a classic actin filament cross-linking protein (128). There are four isoforms of alpha-actinin (1 to 4), each expressed from a different gene. Isoforms 1 and 4 are expressed in non-muscle cells and have molecular weights of approximately 100 kDa on polyacrylamide gels. In contrast, isoforms 2 and 3 are expressed in skeletal and cardiac muscle cells. Solid phase binding experiments between smooth muscle-purified alpha-actinin (specifically from chicken gizzard) and L-selectin peptide revealed that the interaction was specific, and further confirmed by immunoprecipitation (118). Deletion of the C-terminal 11 amino acids of L-selectin (called ‘LΔCyto') abrogates interaction with alpha actinin binding (118). Injection of cell lines expressing LΔCyto L-selectin into the circulation of rats revealed a significant reduction in rolling efficiency within inflamed mesenteric venules (129). These studies strongly imply the requirement for the cytoplasmic tail of L-selectin in regulating rolling interactions. However, little is known about how alpha-actinin interacts with L-selectin and whether the interaction is regulated by (serine/tyrosine) phosphorylation of the L-selectin tail or the production of secondary messengers (e.g., Ca^2+^). Isoforms 1 and 4 of alpha-actinin possess CaM-like EF hands, which can bind Ca^2+^ and inhibit actin cross-linking activity (128). Interestingly, isoforms 1 and 4 expressed in smooth muscle cells (as in chicken gizzard) exist as EF domain splice variants that are Ca^2+^-insensitive (128). As Ca^2+^-sensitive and insensitive forms of alpha-actinin were likely used by Pavalko et al. (118), it is currently unclear if Ca^2+^ binding to alpha-actinin might play an active role in regulating L-selectin binding. Despite its conserved identity with CaM, the EF-hand of alpha-actinin is not believed to interact with L-selectin as the amino acids that support binding of CaM and alpha-actinin are located at opposite ends of the L-selectin tail (130) (see Figure 1A). Non-muscle alpha-actinin is known to bind to other tails of cell adhesion molecules and is reviewed extensively elsewhere (131).

### Calmodulin (CaM)

CaM is an 18 kDa calcium binding protein, which was first identified to bind L-selectin in immunoprecipitation experiments, and subsequently confirmed using *in vitro* solid-phase binding assays (109). CaM binds constitutively to L-selectin in resting cells. Its interaction is thought to render the membrane-proximal cleavage site resistant to proteolytic attack. Upon cell stimulation, CaM dissociates to promote an allosteric change in the cleavage site to drive ectodomain shedding by ADAM17. Serine 364, but not S367, on human L-selectin has been shown to be responsible for the dissociation of CaM from L-selectin (87). Indeed, mutation of S364 to alanine significantly reduces phorbol myristate acetate (PMA)-induced shedding (132). Biophysical experiments show contradictory models for how CaM interacts with L-selectin. One report suggests that CaM binds to both a membrane-proximal region of the L-selectin tail, and a region within the transmembrane domain (133). This arrangement of binding was shown to be calcium-dependent and is thought to act in a ratchet-like mechanism, bringing the cleavage site close to the plasma membrane and displacing some of the transmembrane domain into the cytosol. However, this hypothetical view is deemed thermodynamically unfavorable. Another report used lipid bilayers to address a similar question and found that CaM binds to L-selectin in an “extended conformation,” leaving one domain available for interacting with other binding partners. Biochemical and molecular modeling analyses confirmed that it is possible for the tail of L-selectin accommodate CaM and another protein (Ezrin-Radixin-Moesin—see section below). These experiments corroborated with the aforementioned biophysical data that hypothesized an extended form of CaM could accommodate ERM (134). Some suggestions of how different binding partners could be configured during different stages of the multi-step adhesion cascade is described elsewhere (135). It is still not clear whether CaM binding to the L-selectin tail occurs in a calcium-dependent or independent manner, and experiments have been able to support both views (130, 134).

### Ezrin-Radixin-Moesin (ERM) Proteins

**Figure 2:**
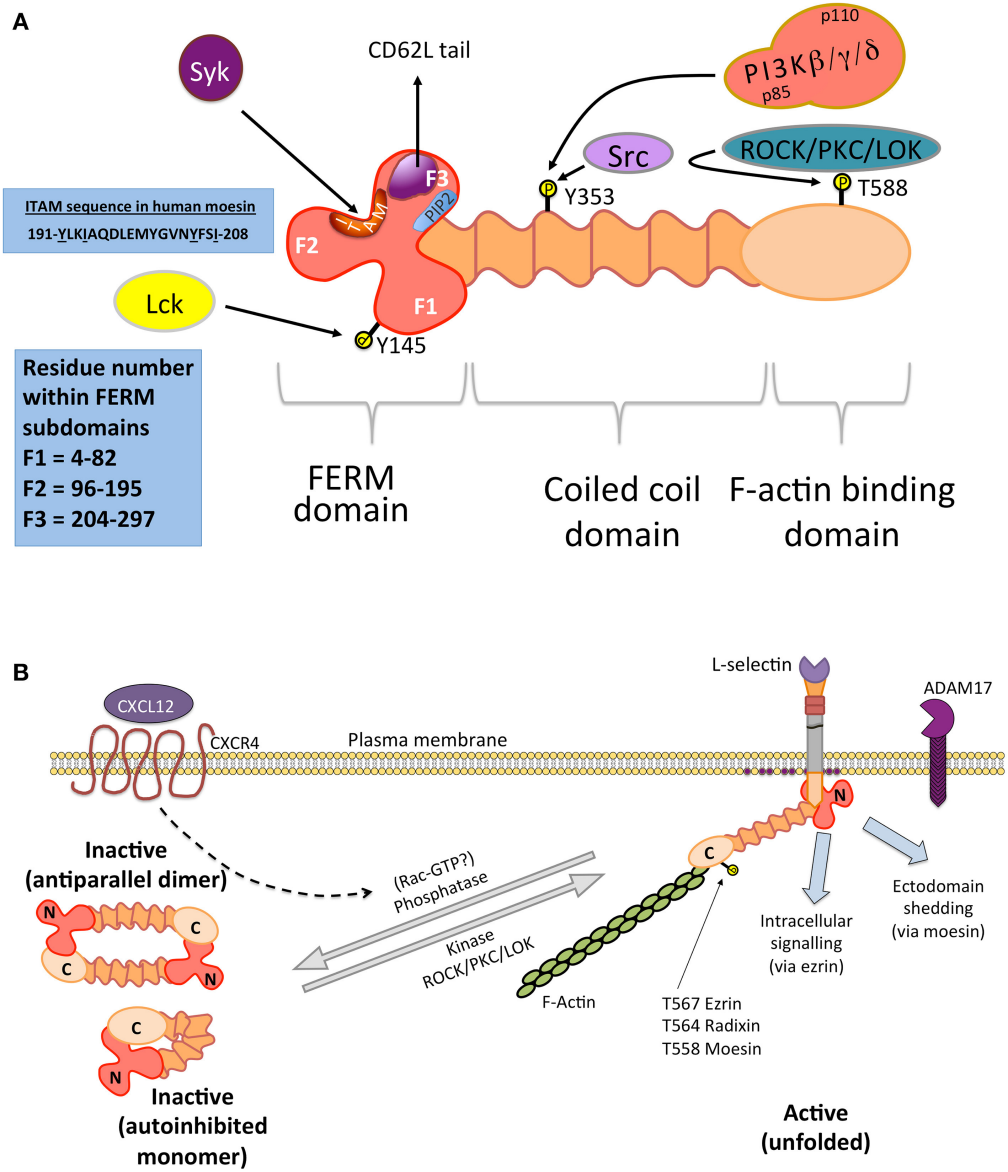
ERM structure and function in immune cells. **(A)** Domain organization of ERM: X-ray crystallographic studies reveal that the N-terminal FERM domain contains a globular clover leaf-shape (136), classified into 3 distinct subdomains (blue box indicates residue numbers that make up each F subdomain). The FERM domain contains multiple interaction sites: a cryptic ITAM (sequence shown), which can recruit Syk, a PIP2 binding site and a region responsible for binding to L-selectin. The tyrosine kinase Lck is responsible for phosphorylating Y145 of the FERM domain in T-cells (137). Src phosphorylation of Y353 in the central coiled-coil domain of ezrin can lead to the recruitment of class I PI3K, via the p85 regulatory subunit (138). Although originally identified in columnar epithelial cells, confocal microscopic colocalisation of PI3K with phospho-Y353-ezrin is witnessed in T-cell receptor-stimulated T-cells (139). These observations suggest that a PI3K/ezrin interaction is possible in leukocytes. **(B)** ERM proteins reside in the cytosol as inactivate parallel homodimers or in an autoinhibited conformation. Binding of ERM to PIP2 within the inner leaflet of the plasma membrane (purple circles) alleviates the inactive conformation, allowing the C-terminal domain to interact with filamentous actin (F-actin). In this manner, ERM are typically classified as membrane/cytoskeleton cross-linkers. The kinase activity of serine/threonine kinases (e.g., ROCK, PKC, or LOK) target a conserved threonine residue in all unfolded ERM (T567 in ezrin, T564 in radixin, and T558 in moesin), stabilizing the open conformation. Recent evidence suggests that ezrin might be involved in regulating intracellular signaling, whereas moesin regulates the clustering of L-selectin prior to its ectodomain shedding by ADAM17 (88). One pathway in which ERM are inactivated has been shown in T-cells, where binding of CXCR4 to CXCL12 leads to the rapid dephosphorylation and microvillar collapse of ERM (140). Although not fully understood, Rac is known to act upstream of the ERM phosphatase (141, 142).

The ERM proteins are a 3-member family of cytoskeletal proteins ranging from 75 to 80 kDa. They all contain very similar domains—a globular N-terminal domain, belonging to the band 4.1 ezrin-radixin-moesin (FERM) superfamily, a central alpha-helical domain and an acidic actin-binding domain (see Figure 2A). The primary role of ERM is to serve as membrane cytoskeleton linkers, however it is becoming increasingly apparent that they play essential roles in mediating signal transduction. In respect of cell adhesion molecules, such as L-selectin, ERM binding restricts their lateral mobility across the plane of the plasma membrane. Anchoring cell adhesion molecules to an underlying cortical actin framework would facilitate receptor clustering and support tethering under flow conditions. Over-expression of L-selectin in fibroblasts can drive the formation of filopodia-like extensions (134). Indeed, ERM proteins are actively involved in forming microvilli (143, 144). ERM adopt an autoinhibited folded conformation when inactive and become “open” when in contact with phosphatidylinositol 4,5-bisphosphate (88, 145–148). Phosphorylation of a conserved threonine residue stabilizes the open conformation (149), which can be targeted by a number of serine/threonine kinases (150–154). Dephosphorylation is triggered by chemokine stimulation and Rac activity in T-cells (140–142) (see Figure 2B), however the mechanism remains poorly understood. Moreover, chemokine induced stimulation of T-cells leads to microvillar collapse that is thought to increase membrane contact between T-cells and endothelial cells.

ERM proteins can also act as adaptors of signal transduction via a number of different pathways (145, 155). In immune cells, ERM possess a cryptic immunoreceptor tyrosine-based activation motif (ITAM) nested within the FERM domain (156–158) (Figure 2A). Studies have shown that the cryptic ITAM can recruit Syk, which in turn can impact numerous downstream pathways—including the activation of integrins toward an “intermediate” state (159, 160). Ezrin is unique from moesin in that it possesses a tyrosine at position 353 (138), which, when phosphorylated by Src, can recruit the p85 regulatory subunit of PI3K. The conversion of phosphatidylinositol (4, 5)-bisphosphate (PIP2) to phosphatidylinositol (3–5)-trisphosphate (PIP3) is essential for the recruitment for guanine nucleotide exchange factors (GEFs—e.g., Vav), which serve to locally activate the Rho family of RhoGTPases, such as Rac (see later in **Figure 4A**).

The ERM proteins were first shown to interact with the L-selectin tail using affinity chromatography, where the 17 amino acid tail was synthesized and immobilized onto sepharose beads (119). Clarified extracts, derived from T-cells stimulated with or without PMA, were passed through these affinity columns to identify novel binders in activated and resting cells. Interestingly, moesin only bound to the column if T-cells were stimulated with PMA. In contrast, ezrin bound to the affinity column in a stimulus-independent manner. These results suggest that ezrin and moesin switch in their binding to L-selectin in a stimulus-dependent manner. Fluorescence Lifetime Imaging Microscopy (FLIM) was used to measure Fluorescence Resonance Energy Transfer (FRET) between L-selectin-GFP and ezrin/moesin-RFP, confirming that L-selectin/ezrin interaction was more readily detectable in resting cells than L-selectin/moesin interaction (88). The biological significance underlying these specific interactions is discussed later.

### Protein Kinase C (PKC) and Serine Phosphorylation of the L-selectin Tail

By tagging the tail of human L-selectin with Glutathione-S-Transferase (GST), Kilian et al. discovered that a 60 kDa PKC-like kinase activity could be precipitated in classic “pull-down” experiments from clarified extracts of Jurkat T-cells (120). Further investigations led them to show that at least 3 isozymes of PKC (α, ι, and θ) are able to phosphorylate the tail of L-selectin. PKC-α and PKC-θ bind more readily to serine phosphorylated L-selectin tail. Given that purified PKC-α can directly phosphorylate serine residues on L-selectin strongly supports the view that PKC is a direct binder of the L-selectin tail. As phosphorylation of S364 drives CaM dissociation (87), it is highly likely that serine phosphorylation of the L-selectin tail occurs during or after shedding. The turnover of the MRF (i.e., the cleavage product of L-selectin that remains in the plasma membrane) has been shown to take ~30 min to be fully degraded (161). There is a potential window of opportunity that the MRF/PKC complex may transduce unique intracellular signals. In support of the MRF/PKC complex, clustering of the T-cell receptor (TCR) has been shown to drive L-selectin shedding in a PKC-dependent manner (162). Importantly, increased retention of PKC onto the tail of L-selectin was observed only after TCR stimulation (120).

Metabolic labeling of cell lines expressing L-selectin with radiolabelled pyrophosphate has shown that S364/S367 are indeed phosphorylated following stimulation with chemoattractants (such as C5a and formyl peptides), IgE or PMA (163). Serine-to-alanine mutagenesis of both serine residues does not show any further transfer of radioactive label to L-selectin, suggesting that Y372 is not phosphorylated—at least in response to the agonists tested in this study.

### AP-1 Adaptin

The binding of L-selectin to μ1A—a 45 kDa protein of the clathrin-coated vesicle AP-1 complex—was identified in classic pull-down experiments and later verified by protein-protein interaction (121). Interestingly, binding was only witnessed in response to PMA-induced cell activation. Although the cellular mechanisms have yet to be defined, it is likely that L-selectin/μ1A interaction drives transport of de novo synthesized L-selectin from the trans golgi network toward the plasma membrane. The PMA-induced mode of interaction might suggest that the MRF is internalized by virtue of μ1A binding, once L-selectin shedding is complete. μ1A may therefore be responsible for sorting the MRF toward endosome-like vesicles for degradation. The cyclical expression of L-selectin in recirculating naïve T-cells may also suggest that, instead of L-selectin shedding, full-length L-selectin may be internalized by μ1A and taken to an endosomal recycling compartment for re-expression back to the plasma membrane at a subsequent stage during lymph node trafficking.

### Indirect Binding Partners of L-selectin

Son of sevenless (SOS)/Grb, p56 Lck and the Src family kinases (Fgr, Hck, Lyn, and Syk) can co-precipitate in anti-L-selectin immunoprecipitates and/or pull-down assays (164–166), but this observation does not confer direct interaction. Given that some of these proteins have already been shown to bind to ERM proteins or CaM (134, 137, 156–158, 167), it is plausible that they form indirect, higher-ordered, complexes with the tail of L-selectin. The cytoplasmic tail of L-selectin is highly basic in nature (e.g., the tail of human L-selectin possesses a predicted pI of 11.1). Based on this property, affinity chromatography experiments (such as pull-down assays) can attract false-positive binders if extracts are not pre-cleared with a scrambled peptide control. Such pre-clearing steps eliminate binders based on charge alone and selectively enrich for sequence-specific binders.

## L-Selectin Clustering by Luminal and Abluminal Ligands

Glycans form a fundamental biological interface between adhering/migrating cells and their immediate microenvironment. Moreover, cell adhesion molecules, such as L-selectin, have the capacity to transduce intracellular signals downstream of glycan binding. There is a consensus of thought that unbound L-selectin is monomeric, which is clustered when bound to glycan (87, 134). This mode of glycan binding drives “outside-in” clustering (and signaling). Inside-out clustering of L-selectin can be driven by other input signals (168, 169). Outside-in L-selectin clustering, facilitated by monoclonal antibodies or the elegant engineering of cytoplasmic tail clustering modules, enhances bond lifetime through increased avidity, which can stabilize and/or decrease rolling speed (170–172). Both outside-in and inside-out modes of L-selectin clustering require association with the underlying actin-based cytoskeleton, which is likely to be through the ERM proteins but this has not been formally tested. How inside-out and outside-in modes of L-selectin clustering inter-relate to one another during a specific cellular process (e.g., TEM) has never been explored.

Given the diversity of L-selectin ligands, and their compartmentalisation within anatomically defined locations within vessel walls, the signals running downstream of L-selectin provides information to the leukocyte's position within the multi-step adhesion cascade (tethering, rolling, adhesion, and transmigration). Whilst L-selectin ligands on the apical aspect of the endothelium have long been characterized as tethering and rolling receptors, the glycans that are enriched in the basolateral aspect, and within the basement membrane, are likely to drive completely different signals. The bond lifetime of L-selectin with apical ligands will be within the order of subseconds [due to rapid catch-slip bond dynamics (104)]. In contrast, L-selectin-dependent adhesion within microenvironments devoid of hydrodynamic shear stress (e.g., within transmigrating pseudopods) will extend from seconds to minutes. The glycan scaffold within regions devoid of shear stress can amplify signaling downstream of L-selectin. Moreover, ectodomain shedding can also fine-tune the magnitude of input signals downstream of L-selectin—limiting signals beyond a critical threshold. In support of this view, clustering L-selectin on Jurkat T-cells can promote ectodomain shedding (173), a phenomenon that can be recapitulated in neutrophils when monoclonal antibodies are used to cluster L-selectin (74). Therefore, understanding the nature of L-selectin ligands is important for appreciating how a changing glycan scaffold can impact leukocyte behavior, for example during transmigration. This section will discuss the nature of luminal and abluminal ligands, providing some insight into how they were identified and characterized.

An excellent in-depth review on the biosynthetic pathways of glycosylation can be found in the following reference (174). Suffice to say, the majority of glycoproteins are subjected to either N- or O-linked glycosylation. N-glycosylation refers to the attachment of glycans to the amine nitrogen of asparagine (N) side chains within the N-X-S/T consensus sequence, where “X” is any amino acid except proline. In O-glycosylation, glycans are linked to the hydroxyl oxygen of serine (Ser) or Threonine (Thr) side chains. Sulfotransferases mediate the covalent attachment of sulfate groups to selected saccharide residues within specific oligosaccharide chains and therefore modulate glycan structure and function.

### Luminal Ligands of L-selectin

The vital role that L-selectin plays in the recirculation of naïve T-cells (46) led researchers to first explore the glycans presented in secondary lymphoid organs. Classic Stamper Woodruff assays (175) revealed that naïve T-cells would accumulate around vessels of thin frozen lymph node sections, which could be functionally blocked by the anti-L-selectin MEL-14 antibody (51). High endothelial venules (HEVs) are specialized microvessels that are unique to secondary lymphoid organs, allowing the entry of blood-borne naïve T-cells into the lymph node parenchyma (in search for antigen priming by resident dendritic cells). The endothelial cells lining HEVs (called high endothelial cells—HECs) can be isolated and grown *in vitro*. Ligands for L-selectin have also been purified and characterized from HEC culture supernatants (176).

**Figure 3:**
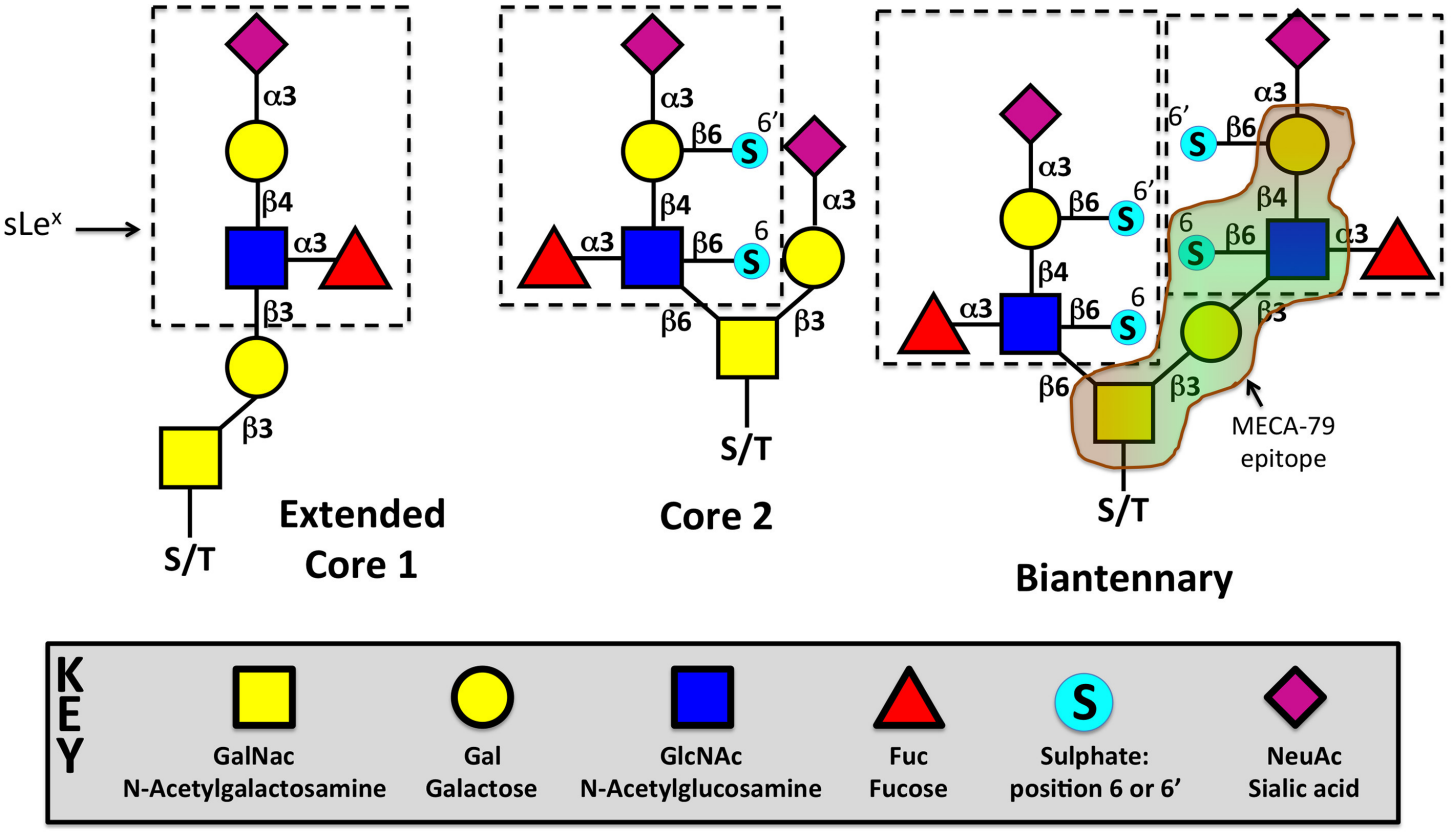
Different glycoforms of O-linked sialyl Lewis X (sLex). sLe^x^ can exist in multiple configurations: extended core 1, core 2, or both (biantennary). Sulfotransferases can attach sulfate esters to Gal or GlcNAc, respectively at positions 6 or 6′. Sulfates can be added to sLe^x^ one or both positions in either extended core 1, core 2, or the biantennary configuration. Recognition of the MECA-79 epitope is provided in the biantennary structure, but can exist in any of the 3 glycoforms. N-linked sLex has also been shown to act as bonafide L-selectin ligands, but are not depicted in this figure. More information can be found in a short concise commentary (177). The glycoforms are drawn in accordance with GlycanBuilder (178). S/T = serine/threonine.

Sialyl Lewis X (sLe^x^) is the minimal structural determinant for luminal ligands of L-selectin, which is composed of sialic acid, galactose, fucose, and N-acetylglucosamine (Siaα2,3Galβ1,4 (Fucα1,3)GlcNAc) (see Figure 3). sLe^x^ is typically O-linked to protein backbones, such as peripheral lymph node addressin (PNAd), CD34, glycosylation-dependent cell adhesion molecule (GlyCAM-1), mucosal vascular addressin cell adhesion molecule-1 (MAdCAM-1), podocalyxin-like protein and spg200 (95, 179–185). Monoclonal antibodies to specific glycan moieties, such as MECA-79 (see below), revealed an apical distribution of these L-selectin ligands in HECs. L-selectin ligands decorating HEV is not solely employed by recirculating naïve T-cells. NK cells are guided to lymph nodes via the same addressin “postcode” to intercept the metastatic spread of B16 melanoma cells to other sites, via local lymph nodes (186, 187). Most recently, neutrophils have been shown to access lymph nodes via the same route as recirculating naïve T-cells, during *Staphylococcus aureus* infection of the lymphatics. The requirement of PNAd (for L-selectin-dependent adhesion) and platelet-derived P-selectin (interacting with neutrophil P-selectin glycoprotein ligand-1—PSGL-1) were equally critical for trafficking to the infected lymph node (188). Since their identification and characterization in secondary lymphoid organs, L-selectin ligands have also been identified in tertiary lymphoid organs surrounding solid tumors (189, 190) and in microvessels within rejecting kidney (191) and heart (192) allografts.

The pan-selectin ligand, PSGL-1, is typically expressed on the surface of leukocytes to mediate a range of complex interactions with other leukocytes, platelets or endothelial cells (193, 194). However, endothelial-specific PSGL-1 expression has been reported in microvessels sprouting into advanced atherosclerotic lesions (68), chronic murine ileitis (195) and benign prostatic hyperplasia (196). These examples of atypical PSGL-1 expression represent unique sites for L-selectin-dependent leukocyte trafficking. PSGL-1/L-selectin interactions were first characterized to support secondary tethering and rolling between bystander leukocytes and adherent leukocytes (197). There is some debate over whether L-selectin ligands are expressed at all in the inflamed post-capillary venules of the cremasteric muscle, and whether L-selectin-dependent recruitment is purely through secondary tethering and rolling on adherent leukocytes or deposited exosome-like structures (79, 198, 199).

sLe^x^ can be linked to lipids, N- or O-linked glycans. The most comprehensively characterized biosynthetic pathway of L-selectin ligands is O-linked sLe^x^, which will be discussed below in further detail. A concise and excellent review on N-linked sLe^x^ is provided in the following review (177).

O-linked sLe^x^ is decorated on protein backbones as single or biantennary branches that are respectively called “core-1 extended” and “core-2” glycans (Figure 3). Both branches extend from N-acetylgalactosamine (GalNac), which itself is covalently attached to a serine/threonine residue by polypeptide N-acetylgalactosamine transferase. sLe^x^ can be modified by the addition of a sulfate ester on C-6 of N-Acetylglucosamine (GlcNAc), which is specific to extended Core 1 branches, or C-6 on Galactose (Gal) of Core 2 branches—respectively termed 6-sulfo or 6'-sulfo (see Figure 3). GlcNAc-6-sulfo is recognized by the MECA-79 monoclonal antibody and is the dominant L-selectin ligand expressed in HEVs and chronically inflamed microvessels (200, 201). Unsulfated sLe^x^ is capable of supporting some lymphocyte homing, as demonstrated in mice lacking the enzymes GlcNAc 6-O-sulfotransferase (GlcNAc6ST)-1 and GlcNAc6ST-2 (201). The core 1 extension enzyme and the core 2 branching enzyme are respectively called: Core1-β3GlcNAcT and Core2GlcNAcT, which have been systematically knocked down in mice to show little impact on lymphocyte homing to lymph nodes *in vivo* (199, 202). Core1-β3GlcNAcT/ Core2GlcNAcT double knockout mice, which lack any detectable MECA-79 expression within HEV, reduced lymphocyte trafficking by only 50%, suggesting other ligands distinct from 6-sulfo sLe^x^ could support lymphocyte recruitment to HEV (203). Mass spectrometry discovered the presence of N-linked 6-sulfo sLex in HEV, which was responsible for the residual lymphocyte recruitment in mice lacking functional O-linked 6-sulfo sLe^x^.

Interestingly, no recruitment of leukocytes to inflamed cremasteric venules was observed in Core2GlcNAcT knockout mice (199). As mentioned before, no detectable ligands for L-selectin are present on endothelial cells of inflamed cremasteric venules (79). It is possible that L-selectin/PSGL-1 secondary tethering and rolling at these sites is highly dependent on Core2GlcNAcT-mediated glycosylation of sLe^x^ onto PSGL-1, which has indeed been reported (204).

### sLex on L-selectin: A Ligand for E-selectin

It has been known for some time that L-selectin is itself decorated by N-linked sLe^x^ (8, 9). This post-translational modification is exclusive to L-selectin in human neutrophils, as mouse neutrophils lack the expression of fucosyl transferase 9 (205). Clustering of L-selectin with E-selectin under flow conditions can drive the co-localization of L-selectin with ADAM17 to the uropod (the rear of the cell), where it is thought that enzyme/substrate coalesce to mediate ectodomain shedding (206). This form of ectodomain shedding is distinct from “mechanical shedding” (75), where neutrophils are perfused exclusively over immobilized sLe^x^ rather than E-selectin (discussed above). E-selectin-mediated clustering of L-selectin on human neutrophils promotes cell priming (i.e., degranulation and superoxide production), via the activation of p38MAPK (207, 208). Two more recent reports have extended our understanding of the molecular mechanisms underlying L-selectin-mediated neutrophil arrest under flow conditions, which have recently been reviewed (209). Perfusion of human neutrophils over immobilized E-selectin leads to the secretion of MRP8 in rolling cells. The secreted MRP8 then binds to TLR4 and promote integrin LFA-1 activation toward an intermediate activation state, driving the transition from rolling to slow rolling on ICAM-1 (210). A separate study showed that L-selectin-dependent adhesion to E-selectin under flow conditions could drive the clustering of L-selectin and transition LFA-1 toward an “active” state (211). Collectively, these studies suggest that signals downstream of L-selectin/E-selectin interaction and PSGL-1/E-selectin interaction converge to arrest neutrophils. Although mouse neutrophils express L-selectin that lack sLe^x^ modification, one study has shown that L-selectin and PSGL-1 co-cluster *in cis* in resting cells, which increases when perfused over P- or E-selectin to activate LFA-1 independently of chemokines (164). It is clear that L-selectin-dependent signaling is important in activating LFA-1 in mouse and human neutrophils, but whether any of the signaling pathways are conserved between species is yet to be determined.

### Abluminal Ligands of L-selectin

It is increasingly appreciated that L-selectin ligands are present in perivascular and extravascular locations (78, 185, 212–215). This itself challenges the previously held view that the role of L-selectin is restricted to early events in the leukocyte adhesion cascade such as tethering and rolling (216), and supports more recent findings that L-selectin may have additional roles beyond luminal interactions (78, 87, 88). The nature of L-selectin/ligand interactions differs from intravascular ligands, as they occur in regions devoid of flow. Early evidence for extravascular L-selectin ligands was demonstrated by presence of the MECA-79 epitope within the abluminal aspect of HEVs in peripheral lymph nodes (185). Furthermore, in HEVs of Peyer's patches, abluminal expression appeared more abundant than luminal expression, alluding to a role for L-selectin within spatially distinct regions of the vessel wall (185).

The majority of identified extravascular ligands are basement membrane proteoglycans, including members of the heparan, dermatan, and chondroitin sulfate proteoglycan families (HSPGs, DSPGs, CSPGs) (212, 215, 217, 218). Using immunohistochemistry in kidney sections, HSPGs were identified as L-selectin ligands, using a chimeric protein consisting of the L-selectin extracellular domain fused to the Fc portion of IgM (L-selectin-IgM). L-selectin-IgM binding was abrogated by heparitinase I but not sialidase treatment (which removes sialic acid from sLe^x^) (212, 213). However, although L-selectin co-localizes with the HSPGs perlecan, agrin, syndecan-4 and collagen type XVIII in the kidney, only collagen type XVIII was responsible for L-selectin binding, which was blocked in collagen XVIII-, but not perlecan-deficient mice (213). Interestingly, despite the presence of collagen type XVIII in some renal structures, L-selectin-IgM was unable to bind in some regions, suggesting that not all collagen XVIII molecules are permissive for L-selectin binding. This indicated that specific GAG chains may be necessary, which was further characterized as requiring O-sulphation (213). Supporting evidence was shown in heparin-mediated inhibition of L-selectin, requiring 6-O-sulphation of the glycolsaminoglycan (GAG) chains in heparin (219). GAG chain length is also an important determinant of L-selectin binding, as *in vitro*, L-selectin binding efficiency was reduced for lower molecular weight heparin relative to full length heparin (213).

Binding of other GAGs to L-selectin has also been demonstrated. In cartilage, L-selectin binding was removed by chondroitinase ABC but not heparitinase I, suggesting the presence of CSPG or DSPGs (213). Similarly, chondoritinase ABC reduced L-selectin binding in particular regions of the kidney, suggesting the contribution of CSPGs or DSPGs. The CSPG, versican, has been identified as an L-selectin ligand in the kidney (212, 220, 221). Like for HSPGs, these interactions depend on particular GAG chains and, similarly, require sulphation (220). Furthermore, the DSPG biglycan on microvascular endothelial cells of the endometrium binds L-selectin (218), and *in vitro* assays have demonstrated the ability of biglycan to induce L-selectin clustering (87). An increasing gradient from the apical to basal aspect of the endothelium has been observed for HSPGs and subsequently for biglycan (87, 222), which may influence L-selectin clustering, and its subsequent shedding, at high ligand densities (173, 223).

Interestingly, whilst binding of L-selectin to sLe^x^ modified vascular ligands occurs optimally at physiological blood pH 7.4, binding to extravascular ligands is optimal at pH 5.6 (224). This could reflect an altered conformation of L-selectin in acidic inflammatory environments that allows binding to different types of ligand, for example by protonation of histidine residues at low pH. Indeed, the CTLD of selectins contain clusters of basic amino acid residues that bind sulfate groups on GAGs via hydrogen bonds (225). At pH 5.6, the CTLD is additionally protonated, increasing the number of H-bonds between the groups (225). Perhaps, under inflammatory or hypoxic conditions in which an acidic environment is generated, the lowering of pH could act as a “switch” for L-selectin binding with greater affinity to subendothelial ligands to facilitate leukocyte tissue infiltration. *In silico* modeling suggests that the sulfate density of the GAG chain is also an important determinant of ligand binding. However, in contrast to Celie et al. who suggest the importance of 6-O-sulphation, here, 3-O-sulfation of HSPGs and 4,6-O-sulfation of CSPGs are reported to increase affinity (225). Whilst the binding site for sulphated proteoglycans is unknown, it seems plausible that binding may involve a cationic region of the CTLD distinct from the sLe^x^ site, as has been shown for sulphated glycolipids (226).

In a rodent model of renal ischaemia-reperfusion (I/R) injury, L-selectin binding to HSPGs was evident in the subendothelial region of interstitial capillaries, which was not detectable in healthy kidneys (214). Mice lacking functional collagen XVIII and perlecan displayed reduced monocyte influx to the kidney 24 h post-I/R injury. Consistent with this, human kidney post-transplant biopsies and acute allograft rejection biopsies showed greater HSPG dependent L-selectin binding to the basement membrane of peritubular capillaries than control kidneys (214). The mechanisms of regulation of HSPG binding of L-selectin are not fully understood, but HSulf 1 and 2 enzymes have been identified in humans to cleave 6-O-sulfate residues (227). In acute allograft rejection, HSulf 1 was downregulated (214), leading to the speculation that HSulf1 may cleave 6-O-sulphated HSPG chains constitutively under basal conditions, but, under inflammatory conditions, its downregulation causes HSPGs to retain 6-O-sulphation and bind L-selectin. The relevance of subendothelial L-selectin ligands may be of importance in inflammatory situations involving endothelial injury where basement membrane proteoglycans are exposed.

### Extravascular Ligands of L-selectin

In addition to L-selectin ligands in luminal and subendothelial environments, putative ligands have been identified in extravascular locations. Using recombinant L-selectin fused to IgG as a histological probe, binding of L-selectin to ligands was identified in myelinated regions of the cerebellum and spinal cord, but not peripheral nervous system, in human, mouse or rat tissues (228, 229). L-selectin ligands were subsequently shown to localize specifically to myelin (230). Ligands were also located in the renal distal tubule, and upon obstruction of the ureter, were found to relocate to peritubular capillaries where infiltration of inflammatory cells ensued (229, 231). Binding of L-selectin-IgG to ligands in the cerebellum and renal distal tubule was abolished by organic solvents and could not be detected by SDS-PAGE, suggesting that they may be glycolipid, rather than protein ligands (229).

Whilst it seems that such ligands would seldom encounter L-selectin due to its shedding from leukocytes during extravasation, incomplete L-selectin shedding, or restoration of surface expression could allow this interaction to initiate an inflammatory response. Indeed, it has been speculated that the interaction between L-selectin on leukocytes and ligands on myelin may provoke an inflammatory response to drive demyelinating diseases (230). This is supported by the finding that L-selectin deficiency prevents macrophage-mediated destruction of myelin in the experimental allergic encephalomyelitis animal model which has clinical similarities to human multiple sclerosis (232). Thus, L-selectin appears to be involved in non-migratory functions. Shedding of L-selectin may serve, in part, to limit the interaction of leukocytes with such extravascular ligands to avoid inappropriate leukocyte activation under normal circumstances. Interestingly, L-selectin is also expressed on trophoblasts and interacts with ligands upregulated on the uterine luminal endothelium during the receptive phase for embryo implantation (233), perhaps performing a role in epithelial “capture” of the blastocyst, analogous to its role in leukocyte capture from blood.

## Non- Canonical Roles for L-Selectin in Regulating Pseudopod Invasion and Protrusion During TEM

Most textbooks will depict ectodomain shedding of L-selectin occurring on leukocytes that have adhered to the apical aspect of the endothelium, following chemokine-dependent integrin activation. Recent evidence in monocytes would suggest that this is clearly not the case. Live imaging of CD14^++^ CD16^−^ classical human monocytes crossing TNF-activated HUVEC under flow conditions led to the observation that L-selectin undergoes ectodomain shedding exclusively during TEM, and not before (87). Suggestions have been proposed that L-selectin is involved in transducing signals (likely co-stimulatory signals alongside integrin- and chemokine-dependent signaling) to drive pseudopod protrusion in TEM. The window within which L-selectin can transduce signals in TEM is limited by its ectodomain shedding, which is maximal when monocytes have fully entered the subendothelial space. In support of L-selectin transducing signals prior to completion of TEM, blocking ectodomain shedding in CD14^++^ CD16^−^ monocytes did not impact the kinetics of TEM per se, but monocytes entering the subendothelial space lost front-back polarity – producing unstable and excessive protrusions.

The molecular mechanism underpinning L-selectin signaling during TEM, whilst difficult to define in primary human monocytes, was determined in cell lines. Genetic engineering of THP-1 (monocyte-like) cells co-expressing L-selectin alongside a known binding partner (e.g., CaM, ezrin, or moesin), tagged to either green/red fluorescent proteins (RFP/GFP), enabled close inspection of protein-protein interactions within transmigrating pseudopods and non-transmigrated uropods. Fluorescence lifetime imaging microscopy (FLIM) was used to quantify Förster resonance energy transfer (FRET) between the GFP and RFP tags to understand the spatial organization of L-selectin with its binding partners within sub-10 nm distances. THP-1 cells captured in mid-TEM revealed that L-selectin interacts with CaM in both transmigrated pseudopods and non-transmigrated uropods (87). As TEM proceeds, CaM dissociates from the L-selectin tail, exclusively within transmigrating pseudopods, driven by phosphorylation of S364. Using similar FLIM/FRET approaches, ezrin and moesin were shown to bind sequentially with L-selectin as TEM proceeds (88). Ezrin binds initially to L-selectin, which is swapped-out by moesin, exclusively within transmigrating pseudopods. The change-over in L-selectin binding from ezrin to moesin is likely to reflect when signaling downstream of L-selectin is halted and ectodomain shedding ensues. Blocking L-selectin shedding leads to the sustained interaction of ezrin with L-selectin, where no exchange with moesin occurs. These outcomes would strongly suggest that it is the L-selectin/ezrin interaction that drives signaling for pseudopod protrusion. Interestingly, knocking out moesin, but not ezrin, in mouse T-cells can increase the surface expression of L-selectin (139, 234). These observations imply that in T-cells moesin acts as a “pro-shedding factor,” but ezrin does not.

### L-selectin-Dependent Signaling During TEM

Unlike the processes of rolling, slow rolling and firm adhesion, little is known about L-selectin-dependent signaling with respect to pseudopod protrusion in TEM. One can speculate that the contribution of luminal, abluminal and interstitial ligands will play a major role in modulating signals downstream of L-selectin/ligand interaction and clustering. Luminal glycoprotein ligands must contain the sLe^x^ glycan core to participate in singular catch bonding of L-selectin, a biomechanical property that is critical for leukocyte capture under physiological shear forces. These ligands are likely to favor the binding of monomeric L-selectin on microvilli. In contrast, the subluminal ligands that bind L-selectin under shear free conditions neither contain nor depend on the sLe^x^ core, and likely share unique highly clustered arrays of sulfates [e.g., in CNS sulfolipids (232)]. These multivalent ligands are therefore specialized in clustering L-selectin and triggering different magnitudes of outside-in signals to facilitate migration or non-migratory processes [e.g., where myelin damage by L-selectin expressing effector T cells proximal to inflamed CNS (232)].

The Kyoto Encyclopedia of Genes and Genomes (KEGG) pathway for human transendothelial migration shows just 4 cell adhesion molecules involved in TEM (https://www.genome.jp/kegg-bin/show_pathway?map=hsa04670&show_description=show), with little insight into the intracellular proteins that mediate this process. In contrast, nearly 20 genes within the leukocyte are required to participate in firm adhesion to the apical aspect of the endothelium. The mechanisms that drive pseudopod protrusion are likely to involve numerous integrin and non-integrin cell adhesion molecules and chemokine (or other G-protein-coupled) receptors. Many reports have documented the contribution of L-selectin in the activation of integrins (164, 211, 235–240), mobilization of chemokine receptors (241–243), and cellular responsiveness to chemokines (65)—all of which play essential roles in TEM. Blocking L-selectin shedding through the expression of ΔM-N L-selectin leads to the sustained and exclusive interaction with ezrin in transmigrated pseudopods. Engineering the R357A mutation [which blocks ERM binding (20, 88)] into ΔM-N L-selectin significantly reduces the multi-pseudopodial extension phenotype, strongly implying that L-selectin/ezrin interaction is driving pseudopod protrusion.

**Figure 4:**
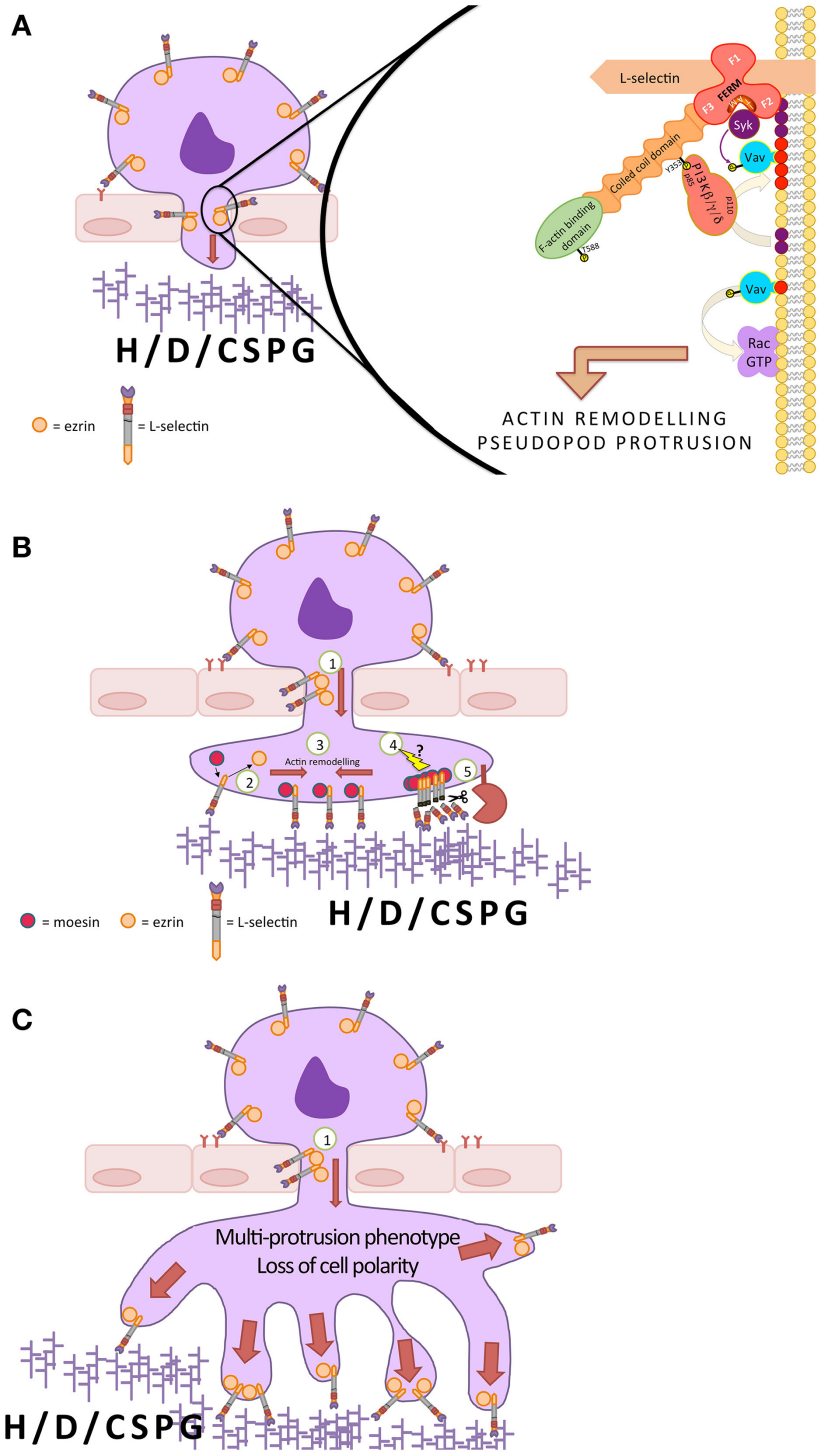
A possible mechanism underlying L-selectin-dependent pseudopod protrusion during monocyte TEM. **(A)** In early monocyte TEM, L-selectin interacts preferentially with ezrin. Moesin and ezrin are the most abundant ERM proteins in monocytes, with little to no expression of radixin. Ezrin can interact with the p85 subunit of class I PI3Ks via phospho-Y353 (a known target of Src kinase). The ezrin-anchored PI3K catalytic subunit (p110) converts PIP2 (purple spheres) into PIP3 (red spheres) within its local vicinity. The most common p110 isoforms in leukocytes are β, δ, and γ. The plekstrin homology domain of guanine nucleotide exchange factors (GEFs), such as Vav, can bind to PIP3 and mediate their effects on the small Rho family of GTPases, such as Rac. The FERM domains of ERM contain a cryptic ITAM, which, upon phosphorylation, can support the binding of spleen tyrosine kinase (Syk). Syk can phosphorylate Vav, increasing its GEF activity significantly toward GTPases like Rac. PI3K and Rac play pivotal roles in pseudopod protrusion, but the molecular mechanism in the context of TEM is currently not known. **(B)** As described in **(A)**, during early TEM, the interaction between L-selectin and ezrin is thought to contribute to pseudopod protrusion (1). As TEM proceeds, the L-selectin within transmigrating pseudopods exchanges affiliation from ezrin to moesin (2). This exchange could happen when L-selectin makes contact with high-density heparan, dermatan or chondroitin sulfate proteoglycan families (H/D/CSPG) within the subendothelial space, such as the basement membrane. Phospho-cycling of serine residues within the L-selectin tail could also be contributing to the exchange mechanism, where phosphorylation drives ezrin dissociation and dephosphorylation allows binding of moesin. The moesin/L-selectin interaction leads to the remodeling of L-selectin into clusters (3), possibly by the remodeling of the cortical actin cytoskeleton. The clustered L-selectin may also trigger as yet unexplored signals (4) that lead to ectodomain shedding by ADAM17 (5). Currently, it is thought that the exchange of ezrin for moesin inhibits further protrusion and rapidly shuts down L-selectin-dependent adhesion in this region by activating shedding. **(C)** Blocking L-selectin shedding in monocytes during TEM sustains the L-selectin/ezrin interaction, which exacerbates the signaling mechanism proposed in **(A)**, leading to the formation of multiple pseudopodial extensions and a loss in cell polarity of fully transmigrated cells.

The events downstream of L-selectin/ERM interaction, in respect of pseudopod protrusion during TEM, remain elusive. Figure 4 proposes a mechanistic model that could link L-selectin to pseudopod protrusion. Ezrin is unique from moesin in that it can interact with the p85 regulatory subunit of PI3K (138). AMC of L-selectin in T-cells can activate the small GTPases Ras and Rac (166), which in turn can promote two inter-linked signals. Rac can target downstream effectors to remodel the actin-based cytoskeleton for pseudopod formation in leukocytes and other cell types (244–247). The catalytic activity of PI3K is significantly enhanced in the presence of activated Ras, resulting in increased local pools of PIP3 production (248, 249). Guanine nucleotide exchange factors (GEFs), such as Vav [a known Rac GEF (250, 251)], is targeted to the plasma membrane via its plextrin homology (PH) domain. Running in parallel, Syk can bind to a cryptic ITAM sequence within the FERM domain of ERM (156–158). The Syk, which is anchored to ERM, can phosphorylate GEFs within its immediate vicinity, such as Vav (252), to increase its GDP-to-GTP exchange activity. Localized Rac activation will promote actin remodeling and pseudopod protrusion during TEM. The displacement of ezrin from L-selectin by moesin would invariably shut down PI3K-mediated PIP3 production and Rac-mediated protrusion. Again, these models are purely speculative and will require further research to expose the true underlying mechanism.

## Concluding Remarks

Since its identification and characterization, the expression of L-selectin has been carefully interrogated in numerous leukocyte subsets. The motivation underlying this type of classification has been to unearth the ever-expanding functional diversity of individual leukocyte subsets, and their specialized roles in health and disease. A major unanswered question in many such immune cell subsets is to understand if there is a direct role for L-selectin signaling in bestowing their unique functionality.

This review also highlights how the expression of non-cleavable L-selectin in one immune cell-type may confer protection from viruses (61), whereas its expression in another cell type could block polarity necessary for migration toward inflammatory targets (40, 87). Therefore, blocking L-selectin shedding as a therapeutic target will require better understanding of the immune cells it will impact in specific disease settings.

It is highly likely that L-selectin acts as a co-receptor to many of the cellular outcomes featured in this review. Fresh insights into how L-selectin signaling synergises/antagonizes chemokine receptor signaling, integrin signaling, and signaling from non-integrin receptors during adhesion and migration is what is now required to further understand how unique signals are propagated under steady-state and pro-inflammatory conditions.

Finally, technological advances in microscopic imaging have for the first time allowed the interrogation of spatio-temporal interactions (within a sub-10 nm resolution) between L-selectin and its cytosolic binding partners during specific cellular events, such as TEM. Moreover, the recent identification of L-selectin contributing to monocyte protrusion during TEM suggests a revised view on how cell adhesion molecules contribute to the multi-step adhesion cascade. It seems that the one-molecule-to-one-step paradigm may not be as strict as once perceived.

## Author Contributions

All authors listed have made a substantial, direct and intellectual contribution to the work, and approved it for publication.

### Conflict of Interest Statement

The authors declare that the research was conducted in the absence of any commercial or financial relationships that could be construed as a potential conflict of interest.
